# Shifting the Scales: Tirzepatide's Breakthrough in Obesity Management

**DOI:** 10.7759/cureus.60545

**Published:** 2024-05-18

**Authors:** Mohammed Sallam, Johan Snygg, Doaa Allam, Rana Kassem

**Affiliations:** 1 Department of Pharmacy, Mediclinic Parkview Hospital, Mediclinic Middle East, Dubai, ARE; 2 Department of Management, School of Business, International American University, Los Angeles, USA; 3 Department of Management, Mediclinic City Hospital, Mediclinic Middle East, Dubai, ARE; 4 Department of Anesthesia and Intensive Care, Sahlgrenska Academy, University of Gothenburg, Gothen‎burg, SWE; 5 Department of Clinical Pharmacy, Queen's University, Belfast, IRL; 6 Department of Management, School of Business, University of Essex‎, Colchester, GBR

**Keywords:** quality of life, weight management, gip and glp-1 receptor agonist, tirzepatide, obesity treatment

## Abstract

Obesity is a complex and chronic condition that presents a significant global health challenge, resulting in a wide range of health problems, such as heart disease, stroke, and diabetes, therefore diminishing the overall well-being of numerous individuals. Conventional treatment options often prove inadequate, providing temporary relief and frequent weight regain. However, a promising medication called tirzepatide has emerged and been approved by the FDA as a groundbreaking solution for managing obesity. Through its unique mechanism of action as a glucose-dependent insulinotropic polypeptide (GIP) and glucagon-like peptide-1 (GLP-1) receptor agonist, as well as its impressive results in clinical trials, tirzepatide has the potential to completely transform the current approach to treating obesity. This editorial provides a quick snap for the clinical implications of tirzepatide, its mechanism of action, effectiveness, safety record, and the challenges it faces, such as insurance coverage and drug shortages. It emphasizes the importance of an integrated treatment approach and highlights the necessity for continuous research and policy backing to enhance accessibility and advance public health outcomes related to weight management.

## Editorial

The global incidence of obesity and its associated complications is steadily increasing at an alarming rate, adversely affecting the health outcomes of more than one billion individuals across the globe [1]. The World Health Organization (WHO) characterizes obesity as accumulating abnormal or excessive fat that threatens one's well-being. The role of multiple factors, particularly the imbalance between energy intake and expenditure, contributes to the onset of obesity [1].

Conventional approaches, such as changing one's lifestyle, adopting a specific diet, engaging in regular physical activity, and using medications, have frequently produced unsatisfactory outcomes [1]. A limited effectiveness and a significant tendency for relapse mark these outcomes. While various anti-obesity drugs have been developed and granted approval, their effectiveness has been limited, and in some cases, safety concerns have resulted in their removal from the market [1].

The recent approval of tirzepatide by the FDA is a significant and potentially revolutionary development in pharmacological treatment for obesity [2]. This medication, which acts as a dual agonist for the glucose-dependent insulinotropic polypeptide (GIP) and glucagon-like peptide-1 (GLP-1) receptors and is administered subcutaneously once weekly, offers a new and innovative approach to tackling the complex factors involved in obesity.

Tirzepatide's mode of action

Tirzepatide stimulates receptors of hormones released by the intestine, specifically GLP-1 and GIP, to diminish hunger and decrease food consumption. This causes the body to stimulate insulin secretion from the pancreas, which inhibits glucagon production - a hormone that raises blood sugar levels. Consequently, blood sugar levels can be efficiently regulated following a meal. Additionally, tirzepatide influences specific chemicals in the brain, leading to decreased appetite, increased energy expenditure, and the prevention of abnormal fat accumulation in areas of the body that typically have minimal fat deposits. Such effects contribute to notable reductions in weight [2].

**Figure 1:**
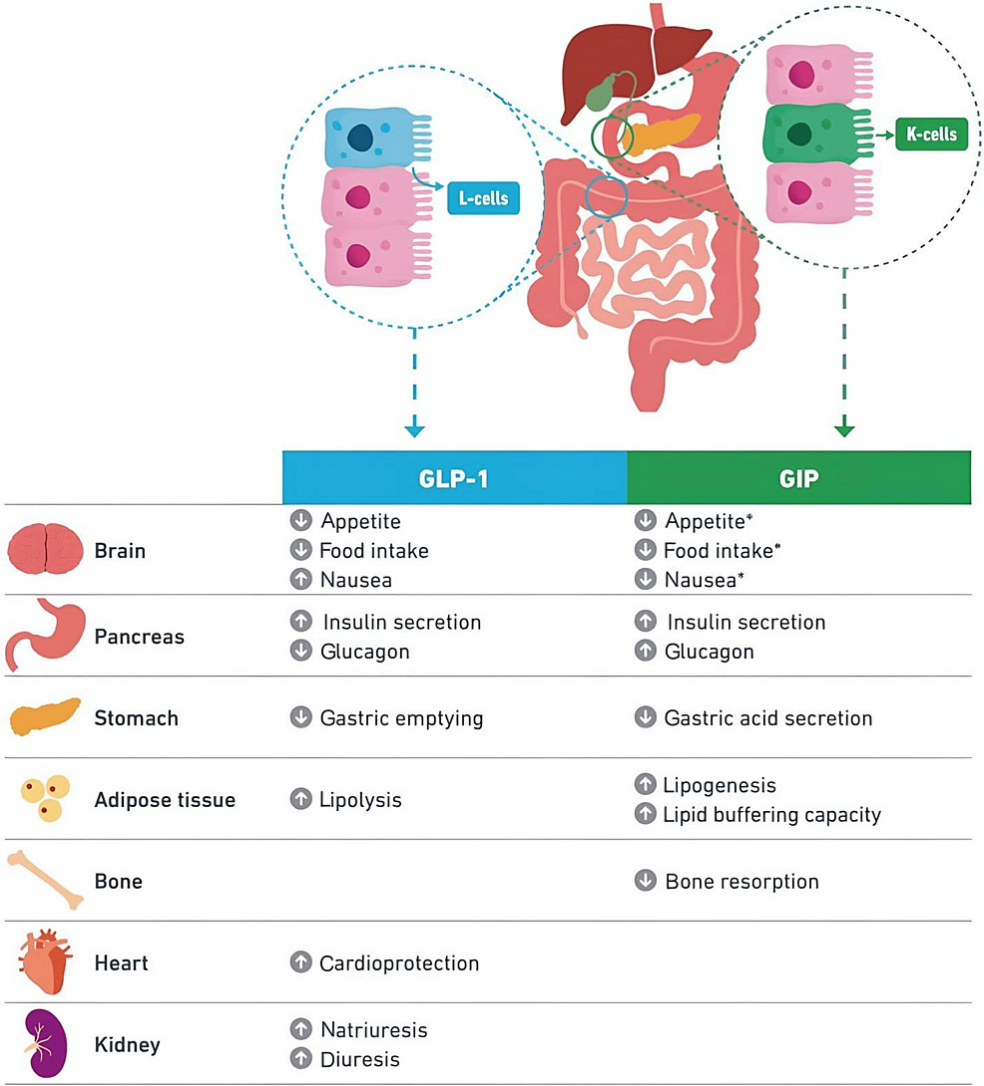
The dual agonist action of tirzepatide on various organs. GLP-1: glucagon-like peptide-1; GIP: glucose-dependent insulinotropic polypeptide. Image adapted from Sinha et al. [2] under the terms of the Creative Commons Attribution Non-Commercial License.

Figure 1 illustrates the multi-dimensional effects of tirzepatide on regulating appetite, glucose metabolism, and fat distribution in the body.

Evaluating clinical efficacy and effectiveness

Clinical trials have generated evidence indicating that tirzepatide proficiently regulates glucose levels and instigates substantial reductions in weight for individuals who are overweight or obese. In contrast to traditional therapies, tirzepatide specifically influences multiple metabolic pathways, enabling it to potentially impact body weight through various mechanisms [2]. A significant decrease in body weight was noticed among individuals with and without type 2 diabetes, indicating a greater level of effectiveness compared to previous records of GLP-1 receptor mono-agonists [2].

The influence of tirzepatide on body weight relies on the dosage, as higher doses often lead to more significant weight reduction. This correlation between dosage and response emphasizes the possibility of customizing tirzepatide according to individual requirements, achieving optimal control of blood sugar levels and weight [2]. It is crucial to observe that tirzepatide should not be regarded as a standalone remedy. It is imperative to adopt a comprehensive approach incorporating a well-balanced diet, consistent physical activity, and lifestyle adjustments to achieve and maintain healthy weight loss [2]. Nevertheless, the distinct capability of tirzepatide to address both blood sugar levels and body weight presents a hopeful path for individuals grappling with obesity-related complications, including type 2 diabetes [2].

Safety profile and tolerability

The safety of tirzepatide plays a pivotal role in its clinical efficacy. The positive gastrointestinal and cardiovascular outcomes observed in studies demonstrate its potential for improved patient acceptance and adherence in the long run [3]. Moreover, the absence of heightened cardiovascular risk is paramount, considering the increasing focus on cardiovascular safety in diabetes management. Although there were reports of gastrointestinal side effects, they were generally mild and temporary. Nevertheless, it is imperative to conduct long-term post-marketing surveillance to understand its safety profile better.

Insurance coverage challenges

The issues surrounding pharmacy insurance coverage for tirzepatide pose considerable obstacles due to its high price and limited coverage from private insurance companies and federal programs. Although attempts are being made to tackle these challenges, the future outlook for tirzepatide insurance coverage remains uncertain. The cost-effectiveness of tripeptide is a significant concern, and it has the potential to greatly impact the healthcare sector's economics.

To ensure the sustainability of the healthcare system and to reverse the rising prevalence of obesity and obesity-related comorbidities, all stakeholders, including pharmaceutical companies, healthcare professionals, and payers, can take steps to encourage effective and equitable treatment options [4].

Challenges in prescribing and drug shortage

Tirzepatide rollout has been hampered by manufacturing shortages, making it difficult for patients to obtain the drug. Physicians are frustrated by the lack of availability, as tirzepatide represents a significant advancement over existing therapies. Many patients have had their prescriptions for tirzepatide delayed or unfilled due to the shortages. Manufacturers must plan and forecast accurately to meet the high demand [5]. Until supply increases, prescribing tirzepatide will remain a challenge for doctors hoping to get this beneficial new medication to the patients who need it most. The drug shortage highlights ongoing issues with scaling up production for this innovative treatment.

Future strategies and recommendations

The introduction of tirzepatide presents a revolutionary opportunity for the management of obesity and its complications. Additional research should be conducted to determine the most effective dosing schedules and criteria for selecting patients to optimize its advantages [2]. Furthermore, it is crucial to thoroughly investigate the impact of tirzepatide on complications associated with diabetes, such as nephropathy and retinopathy, to obtain a comprehensive assessment. Moreover, conducting comparative trials with other emerging therapies and considering real-world evidence will aid in refining its role in the treatment landscape.

Conclusion

Tirzepatide marks a significant advancement in the fight against obesity, a condition that has long posed challenges for public health worldwide. Acting as a dual agonist for GIP and GLP-1 receptors, tirzepatide not only displays potential in reducing weight through its diverse metabolic actions but also aligns with broader actions to address related complications like type 2 diabetes. The introduction of this medication highlights the importance of a comprehensive approach to treating obesity, encompassing sustained lifestyle adjustments alongside pharmacological interventions. It is crucial to effectively incorporate tirzepatide into comprehensive treatment frameworks, guaranteeing accessibility and optimal utilization to maximize its health benefits while effectively managing potential side effects. Through ongoing research, well-considered policymaking, and collaborative healthcare strategies, tripeptide has the potential to greatly enhance the quality of life and mitigate the healthcare burdens associated with obesity.
